# DOF-binding sites additively contribute to guard cell-specificity of *AtMYB60 *promoter

**DOI:** 10.1186/1471-2229-11-162

**Published:** 2011-11-16

**Authors:** Eleonora Cominelli, Massimo Galbiati, Alessandra Albertini, Fabio Fornara, Lucio Conti, George Coupland, Chiara Tonelli

**Affiliations:** 1 Dipartimento di Scienze Biomolecolari e Biotecnologie, Università degli Studi di Milano, Milano, Italy; 2 Fondazione Filarete, Milano, Italy; 3 Max Planck Institute for Plant Breeding Research, Cologne, Germany; 4 Istituto di Biologia e Biotecnologia Agraria, CNR, Milano, Italy; 5 Dipartimento di Biologia, Università degli Studi di Milano, Milano, Italy

## Abstract

**Background:**

We previously demonstrated that the *Arabidopsis thaliana *AtMYB60 protein is an R2R3MYB transcription factor required for stomatal opening. *AtMYB60 *is specifically expressed in guard cells and down-regulated at the transcriptional levels by the phytohormone ABA.

**Results:**

To investigate the molecular mechanisms governing *AtMYB60 *expression, its promoter was dissected through deletion and mutagenesis analyses. By studying different versions of *AtMYB60 *promoter::GUS reporter fusions in transgenic plants we were able to demonstrate a modular organization for the *AtMYB60 *promoter. Particularly we defined: a minimal promoter sufficient to confer guard cell-specific activity to the reporter gene; the distinct roles of different DOF-binding sites organised in a cluster in the minimal promoter in determining guard cell-specific expression; the promoter regions responsible for the enhancement of activity in guard cells; a promoter region responsible for the negative transcriptional regulation by ABA. Moreover from the analysis of single and multiple mutants we could rule out the involvement of a group of DOF proteins, known as CDFs, already characterised for their involvement in flowering time, in the regulation of *AtMYB60 *expression.

**Conclusions:**

These findings shed light on the regulation of gene expression in guard cells and provide new promoter modules as useful tools for manipulating gene expression in guard cells, both for physiological studies and future biotechnological applications.

## Background

Land plants uptake carbon dioxide for photosynthesis and lose water vapour by transpiration through stomatal pores, present on the surface of leaves and stems. The opening and closure of the pore is mediated by turgor-driven volume changes of two surrounding guard cells, whose pressure is dynamically adjusted according to environmental and hormonal cues. In response to abiotic stresses, such as drought or high salinity, one of the most rapid responses of plants is the closure of stomata, mediated by the hormone abscisic acid (ABA), to prevent excessive water loss by transpiration (reviewed in [1]).

The genetic manipulation of stomatal activity is emerging as a promising approach to reduce the water requirement of crops, and to enhance productivity under stress conditions [2]. Proper engineering of stomatal responses requires the use of guard cell-specific promoters, or the identification of guard cell-specific mutants, to avoid undesirable side effects on plant growth and productivity.

Several promoters that confer guard cell-specific gene expression or enhanced gene expression in guard cells have been isolated through different methods: functional characterization of single genes [3-9]; large scale gene- or enhancer-trap screens [10-12]. Moreover transcriptomic and proteomic studies have identified additional candidates [13-16]. Nevertheless the majority of these promoters are not guard cell-specific, as they drive the expression of reporter genes in other cell types, including the vascular tissues [6,10,17,18], flower organs [8,9] or starch containing cells [5], significantly reducing the number of true guard cell-specific full size promoters [3,10,14,19,20]. Most importantly, a detailed experimental analysis of guard cell-specific promoters has been performed only in very few cases [11,12,14].

A true guard cell-specific promoter is driving expression of the Arabidopsis *AtMYB60 *(*At1g08810*) gene [10,19,21,22]. We have previously shown that *AtMYB60 *is expressed in guard cells [10], and the complete 5' and 3' intergenic genomic regions of this gene, cloned respectively upstream and downstream to reporter genes, were able to drive specific expression in guard cells [10,19]. Guard cell specificity of the *AtMYB60 *promoter has been also demonstrated by Nagy *et al*. (2009) and by Meyer et al (2010), who used this promoter to complement the *mrp5-1 *mutant phenotype exclusively in guard cells, and to specifically express the AtLMT12 protein at high levels in guard cells, respectively.

Very little information is available concerning promoter *cis*-elements regulating guard cell-specific expression [8,10-12,14,16]. DOF-binding sites have been suggested to have a role in such a regulation [8,10-12]. DOF (DNA binding with One Finger) proteins are plant specific transcription factors involved in light, phytohormones and pathogen signalling and responses as well as seed development (reviewed by [23]). A role for [T/A]AAAG DOF-binding sites in mediating gene expression in guard cells has been experimentally defined only for the potato *KST1 *gene [8]. However, in Arabidopsis the role of DOF-motifs in controlling guard cell expression is still controversial [10-12]. The study performed on the potato *KST1 *promoter [8] and the bioinformatic analysis performed on several guard-cell specific Arabidopsis promoters [10] suggest that the presence of clusters of DOF *cis*-elements, rather than their absolute number, is important to confer guard cell-specificity to a promoter region [10]. Yet, the role of DOF-binding sites in driving guard cell expression in Arabidopsis and the hypothesis of cluster organization remains to be experimentally investigated.

The guard-cell specific *AtMYB60 *promoter presents several DOF clusters, making it an ideal model to test the hypothesis that DOF clusters are important for guard cell-specific expression. Moreover the *AtMYB60 *expression is modulated by different environmental cues such as light, dark and drought stress [19], suggesting the presence of different *cis*-elements controlling these transcriptional responses. In this report we aimed to isolate the *cis*-elements responsible for the *AtMYB60 *guard cell specific expression. We generated Arabidopsis transgenic lines carrying truncated or mutagenised *AtMYB60 *promoter versions fused to the *GUS *reporter gene. Using a combination of histochemical and expression analysis we were able to identify a minimal promoter necessary and sufficient to drive guard cell specific expression. Using the same tools, we were also able to map a region required for ABA-mediated repression.

## Results

### *In-silico *analysis of the *AtMYB60 *promoter

In a previous study, we demonstrated that the complete 5' and 3' *AtMYB60 *intergenic genomic regions - cloned upstream and downstream of the *β-glucoronidase *(*GUS*) reporter gene, respectively - could specifically drive strong GUS activity in stomata of Arabidopsis seedlings and adult plants [19]. No GUS signals were detected in any other cell type or in tissues devoid of stomata [19].

**Figure 1:**
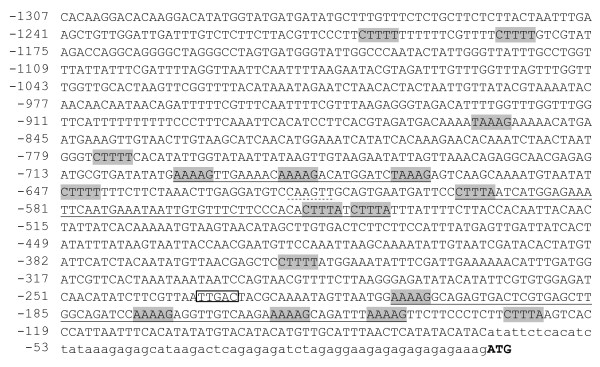
**Nucleotide sequence of the 5'-region of the *AtMYB60 *gene**. Nucleotides are numbered on the left with the translational start site designated as +1. The ATG is in bold. The 5' UTR is in lower case letters. The DOF-binding sites are grey boxed, the W-box, considered in the text, is white boxed. Clusters of DOF-binding sites, as defined by Galbiati and colleagues (2008), are underlined. The CAAGTTG motif described as a putative *cis*-element for ABA repression ([16]) is dotted underlined.

To investigate the possible *cis*-acting elements that regulate *AtMYB60 *expression, we surveyed the genomic region upstream of the *AtMYB60 *translational start codon for the presence of known transcription factor binding sites using the PLACE software [24]. Our analysis produced a significant enrichment in the [A/T]AAAG motifs in the *AtMYB60 *promoter compared to the average distribution of [A/T]AAAG oligos in intergenic regions throughout the Arabidopsis genome (P < 0.01) (Figure 1). Interestingly, these [A/T]AAAG motifs, have been shown to be involved in the regulation of guard cell expression of the potato potassium channel *KST1 *gene [8]. Also, clusters of [A/T]AAAG motifs, required for the binding of DOF-type transcription factors [25], were over represented in different guard cells-specific promoters [6,10,12]. In particular, Galbiati and colleagues suggested, as guard cell-specific *ci*s-element, a cluster of at least three [A/T]AAAG motifs located on the same strand within a region of 100 bp [10]. Using the criteria previously described by Galbiati and collaborators (2008), we found three of these guard cell-specific clusters in the 5' intergenic region of the *AtMYB60 *gene (Figure 1), suggesting a conserved mechanism for guard cell specific expression.

### Identification of the *AtMYB60 *minimal promoter

**Figure 2:**
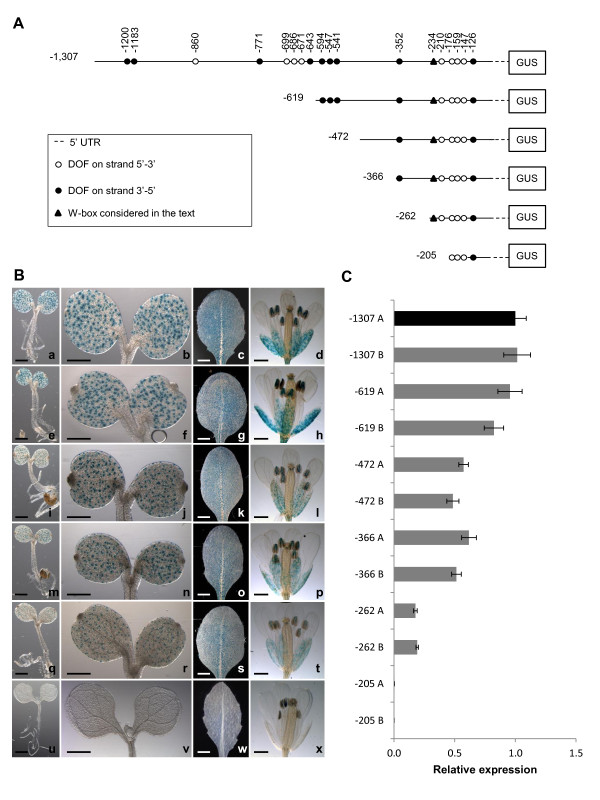
**Deletion analysis of the *AtMYB60 *upstream region**. A, Schematic diagrams of different deletions of *AtMYB60 *upstream region fused to the *GUS *reporter gene. The positions of the different DOF-binding sites and of the W-box, described in the text, are shown. B, Histochemical assay for GUS activity in seedlings, rosette leaves and flowers of plants transformed with -1,307::*GUS *(a-d), -619::*GUS *(e-h), -472::*GUS *(i-l), -366::*GUS *(m-p), -262::*GUS *(q-t) and -205::*GUS *(u-x) constructs. The analysis of independent lines harbouring the same construct showed identical patterns of GUS staining. Samples were incubated in the staining solution for 16 hours for all the lines, with the exception of line -205::*GUS*, for which the staining was prolonged to 48 hours. Scale bars represent 1 mm. C, Relative expression level of the *GUS *reporter gene in the different transgenic lines harbouring the -1,307::*GUS *(-1,307 A and B), -619::*GUS *(-619 A and B), -472::*GUS*, -366::*GUS *(-366 A and B), -262::*GUS *(-262 A and B) or -205::*GUS *(-205 A and B) constructs. Two lines for each construct were analysed by Real Time RT-PCR. The transcript amount in the line -1,307 A was arbitrarily set to 1 (black column) and used to normalize the relative expression levels in each line. The *ACTIN2 *gene (At3g18780) was used as a control.

To gain more insights into the *cis*-elements that regulate the *AtMYB60 *expression in guard cells, we produced a set of Arabidopsis transgenic lines carrying the complete 1,307 bp 5' intergenic region upstream of the translational start codon fused to the reporter *GUS *(construct -1,307::*GUS*, Figure 2A). GUS staining analysis of 15 independent T2 lines revealed that this region contains all the *cis*-acting elements required for expression of the reporter in stomata (Figure 2B), while no GUS signals were detected in any other cell type or in tissues devoid of stomata (Additional file 1).

Next, we made a series of 5' deletions of the -1,307 bp genomic region to define the minimum sequence length required for the expression in guard cells (Figure 2A). These truncated promoters (fused to the *GUS *gene) were stably transferred to Arabidopsis and 10 to 15 independent T2 transgenic lines were analysed in detail. Deletions of the distal part of the 1,307 bp region to position -619 (construct -619::*GUS*), -472 (-472::*GUS*), or -366 (-366::*GUS*) from the ATG codon, did not alter expression of the reporter in guard cells located on both vegetative and floral organs (Figure 2B). Further deletions (to position -262) indicated that the 262 bp proximal region was sufficient to drive expression of the reporter in stomata (Figure 2B). However, the removal of the region between -262 bp and -205 bp (construct -205::*GUS*) completely abolished GUS activity in guard cell (Figure 2B). Transgenic lines carrying the -205::*GUS *fusion did not show GUS staining in any other cell type, even after prolonged staining (up to 48 h, Figure 2B). This finding suggests that the 57 bp region located between positions -262 and -205 contains *cis*-elements essential for expression in stomatal guard cells. Based on these results, we defined the -262 bp region upstream of the ATG codon as the minimal promoter of the *AtMYB60 *gene.

To thoroughly investigate quantitative differences in *GUS *expression among lines carrying different deletion:reporter constructs, we determined the relative amount of *GUS *transcript by quantitative RT-PCR (qRT-PCR). mRNA samples derived from two representative independent lines (A and B) were analysed for each construct (Figure 2C). Lines harbouring the 1,307 bp 5' intergenic region or the -619 deletion fused to the reporter, did not show any significant differences in their *GUS *transcript accumulation. Conversely, deletions to position -472 and -366 resulted in a two-fold decrease in *GUS *expression compared to the -1,307::*GUS *line, while deletion to position -262 resulted in a five-fold decrease (Figure 2C, p < 0.01). These results indicate that one or more sequences with function of enhancer are present in the genomic region between -619 bp and -472 bp and between -472 and -262 from the ATG of *AtMYB60*. In accordance with the results obtained from the histochemical analysis, qRT-PCR experiments did not detect significant *GUS *transcripts accumulation in lines carrying the -205::*GUS *fusion.

### Site-directed mutagenesis of the *AtMYB60 *minimal promoter

**Figure 3:**
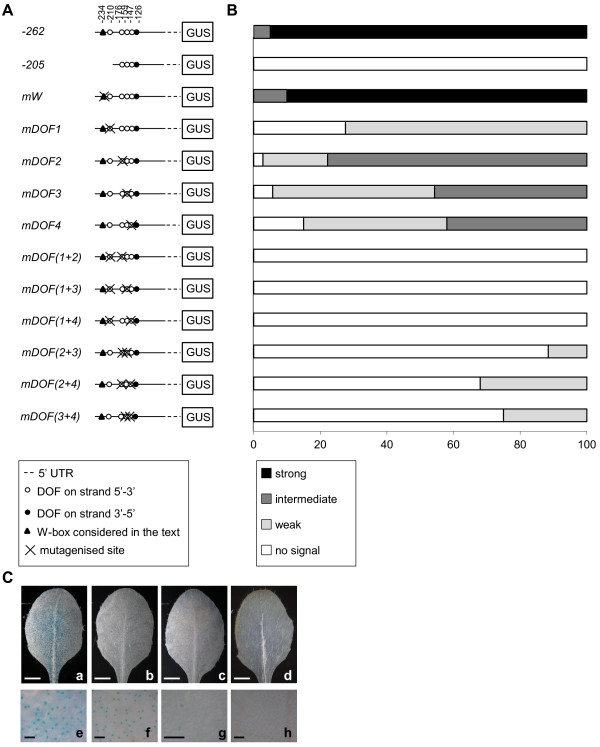
**Role of DOF-binding sites in the minimal promoter in driving GUS activity in guard cells**. A, Schematic diagrams of constructs -262::*GUS*, -205::*GUS *and of constructs containing mutagenised version of the minimal promoter in different DOF-binding sites and in the W-box at position -234. B, Percentage of lines for each construct showing strong (column segment in black), intermediate (in dark grey), weak (light grey) or no signal (white). C, A leaf from a line harbouring the -262::*GUS *construct (a and a particular in e), shown as an example of strong GUS activity. In the following pictures examples of different lines harbouring the *mDOF3*::*GUS *construct showing respectively an intermediate (b and f), a weak (c and g) and no GUS activity (d and h). Scale bars represent 1 mm (a-d) or 0.1 mm (e-h).

Promoter deletion experiments indicate that the *AtMYB60 *minimal promoter region (construct -262::*GUS*) contains all the *cis*-acting elements required to sustain expression of a reporter gene in guard cells. This region encompasses the [A/T]AAAG cluster proximal to the ATG codon, which consists of four AAAAG DOF-binding sites (Figures 1 and 3A). In addition, the PLACE software identified in this region a single W-box, corresponding to the binding site of WRKY transcription factors [26], located upstream of the [A/T]AAAG cluster (Figure 3A). To address the functional significance of the individual *cis*-elements present in the *AtMYB60 *minimal promoter, we evaluated the effects of targeted nucleotide substitutions on *GUS *expression (Figure 3A). Mutated versions of the minimal promoter were generated by PCR and fused to *GUS *and at least 30 T2 independent transgenic lines for each mutated promoter::*GUS *combination were visually scored and classified to reflect their relative guard-cell specific GUS staining. A representative example of each category is provided in Figure 3C.

**Figure 4:**
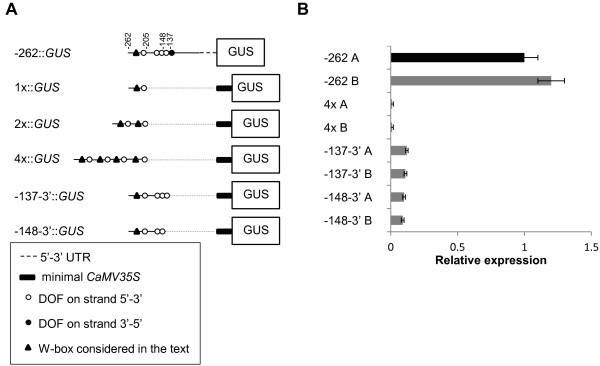
**Oligomerisation of the 57 bp sequence and 3' deletions of the *AtMYB60 *minimal promoter**. A, Schematic diagrams of the constructs. In the 4x::*GUS *construct the fragment of 57 bp between -262 and -205 in tandem array of four copies was fused to the minimal promoter CaMV 35S (min 35S in the scheme, portion between -46 and +1) upstream of the *GUS *reporter gene. In the constructs -137-3'::*GUS *and -148-3'::*GUS*, 3' deleted versions of the *AtMYB60 *minimal promoter were fused to the same portion of the CaMV 35S. (B) Relative expression level of the *GUS *reporter gene in the different transgenic lines harbouring the constructs. Two lines for each construct were analysed by Real Time RT-PCR. The transcript amount in the line -1307 A was arbitrarily set to 1 (black column) and used to normalize the relative expression levels in each line. The *ACTIN2 *gene was used as a control. The symbols are the same described in Figure 2. The dotted lines indicate the regions deleted in *AtMYB60 *minimal promoter sequences.

We initially tested the role of the single W-box *cis*-element, by replacing the consensus sequence TTGAC, with the non-functional TTGAA motif [27]. Lines carrying the mutated W-box (*mW::GUS*) showed similar levels of *GUS *expression to the wild-type promoter, indicating that W-box does not contribute to mediate gene expression in guard cells (Figure 3B). Next, we produced mutant promoters in which single DOF motifs within the [A/T]AAAG cluster were converted to the unrelated CGCGA sequence. Inactivation of the most distal AAAAG site relative to the ATG (hereinafter referred to as *DOF1*) resulted in a dramatic decrease of *GUS *expression (*mDOF1*::*GUS *construct, Figure 4B). 30% of the lines carrying the *mDOF1*::*GUS *construct did not show *GUS *expression, whereas the remaining 70% only showed weak staining, thus indicating a crucial role for *DOF1 *in regulating *AtMYB60 *expression in guard cells (Figure 3B). Mutations of the second, third or fourth most proximal AAAAG site (hereinafter referred to as *DOF2*, *DOF3 *and *DOF4*, respectively), resulted in a reduced *GUS *expression, although to a lesser extent than the one in the *DOF1 *(Figure 4B, m*DOF2::GUS*, *mDOF3::GUS *and *mDOF4::GUS *plants). In particular, none of the 30 *mDOF2::GUS *transgenic lines displayed strong expression of the reporter, nearly 70% showed intermediate expression, 25% showed weak expression and the remaining 5% did not show any GUS staining (Figure 3B). A comparable distribution among strong, intermediate and weak lines was obtained from the analysis of the *mDOF3::GUS *and *mDOF4::GUS *plants (Figure 3B).

To establish whether DOF-binding sites could exert additive roles in mediating gene expression in stomata we produced a second series of promoters, in which two AAAAG motifs were mutated simultaneously. Mutations of *DOF1 *and *DOF2 *(*mDOF(1+2)::GUS*), *DOF1 *and *DOF3 *(*mDOF(1+3)::GUS*) or *DOF1 *and *DOF4 *(*mDOF(1+4)::GUS*) completely inactivated the minimal promoter, as *GUS *expression was abolished in all the *mDOF(1+2)::GUS*, *mDOF(1+3)::GUS *and *mDOF(1+4)::GUS *lines analysed (Figure 3B). Interestingly, the concurrent mutation of *DOF2 *and *DOF3 *(*mDOF(2+3)::GUS*) resulted in a strong, but yet not complete, inactivation of the promoter activity in guard cells, as 15% of the *mDOF(2+3)::GUS *lines displayed weak expression of the reporter in stomata. Likewise, concomitant inactivation of either *DOF2 *and *DOF4*, or *DOF3 *and *DOF4 *did not completely eliminate *GUS *expression in guard cell (Figure 3B). Taken together, these results indicate that the putative [A/T]AAAG DOF-binding sites located in the *AtMYB60 *promoter are necessary to mediate its expression in guard cells.

### A single DOF cluster is sufficient to drive low expression in guard cell

Our deletion analysis of the *AtMYB60 *promoter indicates that the 57 bp region between positions -262 and -205 is essential for gene expression in stomatal guard cells (Figure 2). This region contains the *DOF1 cis*-element required for guard cell expression as shown by mutagenesis analysis results (Figure 3). To establish whether this 57 bp region was sufficient to activate expression in guard cells, we fused one (1x::*GUS *construct), two (2x::*GUS*) and four tandem copies (4x::*GUS*) of the 57 bp fragment to the minimal *CaMV35S *promoter [28] upstream of the *GUS *reporter gene (Figure 4A), effectively reconstructing an artificial DOF cluster containing one, two or four copies of the *DOF1 *element. However, we did not observe GUS activity in any of the 30 independent stable transformants produced for each construct, even after prolonged staining (data not shown). These data were confirmed by qRT-PCR analysis of independent lines carrying the 4x::*GUS *fusion (Figure 4B), indicating that the multimerisation of the *DOF1 *site *per se *is not sufficient to drive gene expression in guard cell. This might derive from an inappropriate organization and/or spatial distribution of the different DOF elements in the context of the minimal promoter. To test this hypothesis we made two 3' deletions of the *AtMYB60 *minimal promoter: the -148-3'::*GUS *and -137-3'::*GUS *constructs containing the first three and four DOF-binding sites respectively of the most proximal cluster fused upstream of the minimal *CaMV35S *promoter (Figure 4B). Our initial histochemical analysis did not reveal any GUS positive lines (data not shown). To substantiate this result we also performed a qRT-PCR analysis on fifteen independent lines for each construct. Interestingly, eight lines out of fifteen showed a low but significant *GUS *transcript accumulation compared to the full length minimal promoter (Figure 4B). These results suggest that the presence of the cluster containing three or four DOF-binding sites is sufficient to drive GUS activity in guard cells, even though at a very low level. This finding implies that other *cis*-elements present downstream of position -137 are required for the full functionality of the minimal promoter.

### The guard cell-related CDF1, CDF2, CDF3 and CDF5 DOF-type transcription factors do not regulate *AtMYB60 *expression in stomata

Target mutagenesis experiments of the *AtMYB60 *promoter demonstrated that [A/T]AAAG DNA consensus motifs are essential *cis*-acting elements in the regulation of *AtMYB60 *expression in guard cells. Consequently, their cognate DOF proteins represent the most likely candidates as *trans*-acting factors. As the Arabidopsis genome contains 36 DOF-coding genes [23], candidate DOF transcription factors involved in the regulation of *AtMYB60 *expression should fulfil two criteria: they should be expressed in guard cells and the loss of their gene function should abolish or significantly down-regulate the expression of *AtMYB60 *in this cell type.

The *CYCLING DOF FACTOR 1 *(*CDF1, At5g62430*) gene, involved in the regulation of photoperiodic flowering, has been shown to be highly expressed in the vascular tissue and guard cells [29]. We thus investigated the expression of the *AtMYB60 *gene in the loss-of-function *cdf1-R *allele. As shown in Additional file 2 we did not detect significant differences in the accumulation of *AtMYB60 *transcripts in homozygous *cdf1-R *plants compared with the wild type.

It is important to note that in photoperiodic flowering, CDF1 acts redundantly with three other DOF proteins, namely CDF2 (At5g39660), CDF3 (At3g47500) and CDF5 (At1g69570) [30], belonging to the same phylogenetic group II [31]. Similarly to *CDF1*, promoter::*GUS *analyses revealed that *CDF2*, *CDF3 *and *CDF5 *are strongly expressed in guard cells.

We thus analysed the expression of *AtMYB60 *in single, double, triple and quadruple *cdf *mutants to determine the possible role of these additional candidate CDF proteins. As for *cdf1-R *mutant, the level of expression of *AtMYB60 *was not significantly reduced in the *cdf2-1*, *cdf3-1 *and *cdf5-1 *single mutants (Additional file 2). Likewise, *AtMYB60 *expression was not altered in any of the double, triple or quadruple mutant combinations, indicating that, despite their expression in guard cells, these four CDF proteins are not *trans*-regulators of *AtMYB60 *expression in stomata (Additional file 2).

### Identification of a promoter region that negatively responds to ABA

**Figure 5:**
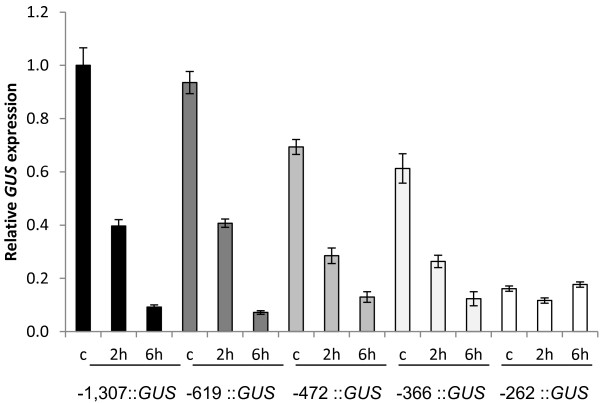
**Expression of the *GUS *gene in different transgenic lines in response to ABA treatment**. Two lines for each construct shown in Figure 2 were analysed by Real Time RT-PCR. c represent the control samples. The transcript amount in the sample -1307 A control was arbitrarily set to 1 and used to normalize the relative expression levels in each line. The *ACTIN2 *gene was used as a control.

We previously reported that transcript accumulation of the *AtMYB60 *gene is rapidly down-regulated by exogenous applications of the hormone ABA, which plays a fundamental role in regulating gene expression in response to drought stress [19]. To identify the promoter region responsible for the ABA-mediated *AtMYB60 *down-regulation, we applied ABA to the previously described transgenic lines harbouring serial deletions of the *AtMYB60 *promoter (Figure 2). Quantitative RT-PCR analysis revealed a similar decrease in *GUS *transcript levels in transgenic lines carrying the full length as well as the -619, -472 and -366::*GUS *fusions (Figure 5). The kinetic of down-regulation of the *GUS *transcript was comparable to the one observed for the endogenous gene *AtMYB60 *[19], indicating that -619, -472 and -366 promoters maintain the sequences responsible for transcriptional down-regulation by ABA. Also, these results suggest that the CAAGTTG motif, present in the *AtMYB60 *promoter between -619 and -613 (dotted underlined in Figure 1), and recently described as overrepresented in ABA-repressed genes [16], does not play a significant role in the ABA-dependent repression of *AtMYB60 *expression. Rather, qRT-PCR experiments performed on different independent lines carrying the -246::*GUS *construct showed that the minimal promoter sequence lacks the region responsible for negative regulation by ABA, as these lines did not show changes in *GUS *expression in response to the hormone as shown in Figure 5.

Taken together these data indicate that, although the minimal promoter maintains the *cis*-elements necessary for guard cell expression, it lacks the motifs that mediate the negative regulation by ABA, becoming ABA-insensitive. We can thus conclude that the region between -366 and -262 contains elements necessary for ABA down-regulation.

## Discussion

Very few guard cell-specific promoters have been described to date [3,10,14,19,20]. Independent studies demonstrated that the *AtMYB60 *promoter can be considered guard cell-specific, being sufficient to drive expression of reporter genes specifically in guard cells [19,21]. Moreover this promoter has also been used to complement a mutant phenotype specifically in guard cells [21], and to investigate subcellular localization exclusively in guard cells [22]. In this study we identified the *AtMYB60 *minimal promoter that is necessary and sufficient to drive guard cell-specific expression.

### DOF-binding sites are required for *AtMYB60 *guard-cells expression

Our *in silico *analysis identified three DOF site clusters (Figure 1). Initial deletion studies revealed a prominent role for the most proximal DOF cluster (relative to the ATG start codon). Site-directed mutagenesis showed that the distal most DOF-binding site (*DOF1 *at position -210, Figure 3) plays a major role in driving guard cell expression compared to other DOF motifs of the same cluster (*DOF2 *at position -176, *DOF3 *at -159 and *DOF4 *at -147, Figure 3). These other DOF elements play partially additive roles, as clearly demonstrated by the combined mutagenesis of these sites and *DOF1 *site which resulted in a drastically reduced GUS activity (Figure 3). DOF-binding sites are thus key determinants in mediating guard cell expression, in accordance with the DOF cluster hypothesis we previously formulated [10]. A suggestion for a similar involvement of DOF *cis*-elements in Arabidopsis derives from the work of Gardner and colleagues (2009) that identified DOF motifs in a region controlling guard cell expression. Other authors identified a region enriched in DOF-binding sites in the guard cell-specific pGC1 promoter, although the mutation of a single DOF site did not impair promoter activity [14]. Interestingly, a DOF cluster organization is present in the promoter of the grape *VvMYB60 *gene, a putative ortholog of *AtMYB60*, indicating a conservation of the cluster structure during the evolution among *AtMYB60 *orthologs [32]. The results reported by Plesch and colleagues (2001) on the DOF motif organisation in the potato *KST1 *promoter highlight a more general evolutionary conservation of this module in the control of guard cell-specific activity of promoters.

Although we cannot rule out the possibility that other unknown transcription factors might interact with those same *cis*-elements, DOF factors represent likely candidates as *AtMYB60 *regulators. The most parsimonious hypothesis resulting from combining our results indicates that DOF proteins act as positive regulators of *AtMYB60*. The potato StDOF1 protein has been shown to bind *in vitro *to the guard cell specific promoter of *KST1 *[8], while no data are available for any Arabidopsis DOF proteins. Among the Arabidopsis *DOF *genes, *CDF1*, *CDF2*, *CDF3*, and *CDF5 *(CDFs) are expressed in guard cells [29]. However, singles and multiple *cdf *mutants show a wild-type pattern of *AtMYB60 *expression, ruling out their involvement in *AtMYB60 *regulation (Additional file 2). The majority of Arabidopsis *DOF *genes are expressed in guard cells [33,34] and may thus act redundantly, as already demonstrated among members of this family [30]. All these aspects do not facilitate the identification of obvious candidates as *AtMYB60 *regulators. We are trying to identify the *DOF *genes involved in the regulation of *AtMYB60 *by analysis of its expression in mutants of genes preferentially expressed in the guard cells (http://bbc.botany.utoronto.ca/efp/cgi-bin/efpWeb.cgi[33]).

### Multiple *cis*-elements participate to enhance *AtMYB60 *guard-cells expression

Transcriptional *GUS *fusions, harbouring different deletions of the 5' intergenic region to position -262 from the ATG, conferred GUS activity exclusively in guard cells (Figures 2 and Additional file 1). The activity of these promoter regions is in apparent discrepancy with the detection of *AtMYB60 *gene expression in seeds, as revealed by available microarray analysis data [33,34] and in roots, as recently reported [35]. One hypothesis to explain this incongruity could be the presence of other regulatory regions present outside the complete 5' and 3' intergenic regions flanking the *AtMYB60 *coding sequence. Intron sequences, for example, may be involved in such a regulation, as previously demonstrated for different plant genes ([36] and references herein).

While guard-cell specific expression was invariably maintained by functional *AtMYB60 *promoter variants, the levels of expression varied considerably. In addition to DOF-binding sites, other *cis*-elements are required to boost the *AtMYB60 *expression. Indeed, an artificial *DOF1 *binding site repeated in single or multiple copies could not drive guard cell expression (Figure 4A). The incorporation of the entire proximal DOF cluster (e.g. -137-3'::*GUS*) resulted in a small but significant guard cell transcriptional activity. Thus, other *cis*-elements downstream of position -137 are required for full activity of the minimal promoter. It is known that *cis*-elements other than DOF-binding sites are involved in the regulation of guard cell expression. In the case of the guard cell-specific *AtPDR3 *gene no [A/T]AAAG clusters were identified in a 1000-bp region upstream of the ATG codon, suggesting the presence of other regulatory units [10].

### Modular organization of the *AtMYB60 *promoter

**Figure 6:**
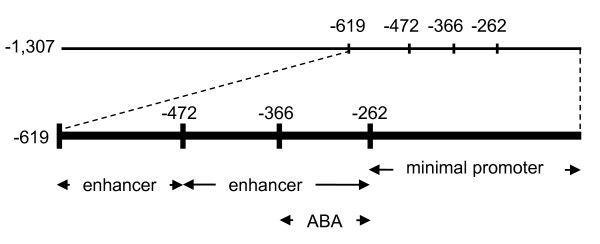
**Modular organization of the *AtMYB60 *promoter**. Different portions of the *AtMYB60 *promoter defined through deletion analysis are shown. ABA indicates the region responsible for the negative regulation by ABA treatment.

In this study we also investigated the regulation of the *AtMYB60 *promoter activity in response to ABA. ABA treatments induce global changes in gene expression in Arabidopsis [16,37-40]. Transcriptomic analyses revealed extensive regulation of gene expression by ABA also in guard cells [13,14,16]. While *cis*-elements that positively regulate the response to ABA have been functionally characterised (for a review, see [41]), those that negatively regulate the response to ABA are largely unknown. A CAA[G/C]TTG motif has been shown to be over-represented in ABA-repressed gene promoters and thus proposed for such a role [16,39]. The *AtMYB60 *promoter contains one CAAGTTG motif between -619 and -613 from the ATG, yet our results do not support its proposed role as negative regulator of ABA response. Conversely, a region between positions -366 to -262 contained the entire requirement for the ABA-mediated repression Figures 5 and 6. It has been proposed that evolution may have favoured the differentiation of mechanisms for ABA down-regulation rather than up-regulation, rendering more difficult for any ABA-repression motif to achieve statistical significance [16]. Our data may provide a valuable model system to clarify the mechanism mediating ABA repression.

Our data suggests a modular organization for the *AtMYB60 *promoter as summarised in Figure 6. Through a serial deletion analysis, we defined the *AtMYB60 *minimal promoter, sufficient to induce guard cell-specific activity (construct -262::*GUS*, Figure 2). A 57 bp region, located between position -262 and position -205, is necessary to confer GUS activity in guard cells (Figure 2A). We also identified two regions that enhance the expression of the *GUS *gene between -619 bp and -472 bp and between -472 and -262 (Figure 2B and 2C). Besides providing pieces of evidence for such modular organization, our work indicates that the different portions of the *AtMYB60 *promoter may prove useful for manipulating gene expression in guard cells, with the possibility to obtain different level of expression. Moreover, the minimal promoter (whose activity is not influenced by ABA) can be used for ABA-independent expression of target genes in guard cells

Interestingly, both the full length and the minimal promoters maintain their guard cell-specific activity in heterologous systems, such as the crop species tomato and tobacco (Francia, personal communication), thus indicating the conservation of this cell-specific regulatory mechanism among different plant species. Moreover, preliminary results suggest that the *AtMYB60 *minimal promoter can be combined with other *cis*-regulatory modules to produce functional guard cell-specific chimeric promoters (Francia, personal communication). As a whole our data demonstrate that both the full length and the minimal *AtMYB60 *promoters provide a valuable tool to manipulate gene expression specifically in guard cells, both for physiological studies and downstream biotechnological applications.

## Conclusions

Our work provides strong evidence for the involvement of [A/T]AAAG elements in the regulation of the *AtMYB60 *expression, illustrating their functional cluster organization. Future work will concentrate on the analysis of candidate DOF transcription factors that control this mechanism. Finally we identify a region of the *AtMYB60 *promoter required for the negative regulation by ABA, offering the possibility to discover novel *cis*-elements for this kind of regulation.

## Methods

### Plant Material

All plant material described was in the Col-0 accession. The *cdf1-R *line (35S::CDF1-RNAi #23) was kindly provided by Takato Imaizumi [29]. The *cdf2-1*, *cdf3-1 *and *cdf5-1 *null alleles are T-DNA insertion line. Single, double, triple and quadruple *cdf *mutants have been previously described [30].

### Construction of *AtMYB60 *promoter::*GUS *fusions

5'-deletions of the 5' intergenic genomic region upstream of the *AtMYB60 *gene were generated by PCR amplification from plasmid p1.3-2.2::*GUS*, previously described [19], using different forward primers and a single reverse primer. Forward and reverse primers incorporated a *Hind*III and a *Bam*HI, respectively. The PCR fragments were cloned into the pCR4-TOPO vector (Invitrogen Corporation, Carlsbad, CA), cut with *Hin*dIII and *Bam*HI and ligated upstream of the *uidA *coding sequence in the pBI101.3 binary vector (Clontech, Palo Alto, CA, USA). The resulting plasmids were renamed -1307::*GUS*, -619::*GUS*, -472::*GUS*, -366::*GUS*, -262::*GUS *and -205::*GUS *(Figure 2).

Chimeric promoters containing different 3'-deleted fragments of the *AtMYB60 *minimal promoter and 46-bp CaMV 35S promoter were produced by amplifying the sequence of the CaMV 35S promoter from -46 to +1 [28] from plasmid pBI121 (Clontech, Palo Alto, CA, USA), using the forward primer 35SXba containing a *Xba*I site and the reverse primer 35SBam with a *Bam*HI site. The PCR product was cloned into the pCR4-TOPO vector and the *Xba*I-*Bam*HI fragment was cloned into the pBI101.3 vector (renamed 35Smin-pBI101.3). The regions from -262 to -137 and from -262 to -148 of the *AtMYB60 *minimal promoter were amplified by PCR from plasmid p1.3-2.2::*GUS*, using the reverse primers p60R6 and p60R7 incorporating a *Xba*I site and a single forward primer p60F3 with the *Hin*dIII site. The corresponding PCR products were cloned into the pCR4-TOPO vector and the *Hin*dIII-*Xba*I fragments were cloned into the 35Smin-pBI101.3 vector to give the -137-3'::*GUS *and -148-3'::*GUS *vectors, respectively (Figure 3).

Chimeric promoters containing different copies of the region between -262 and -205 of the *AtMYB60 *promoter were obtained by synthesising one copy of this sequence, using the forward primer p60F3 with a *Hin*dIII site and the reverse primer p60R3 with an *Xba*I site. The resulting PCR product was cloned into the pCR4-TOPO vector and the *Hin*dIII-*Xba*I fragment was ligated into the 35Smin-pBI101.3 vector (construct 1x::*GUS*). A second copy of this region was generated using the primers p60F3 and p60R5b, both incorporating a *Hin*dIII site; the fragment *Hin*dIII-*Hin*dIII was cloned into the construct 1x::*GUS*, generating the construct 2x::*GUS*. This plasmid was used as a template to generate two other copies of the sequence from -262 to -205 using the primers p60F11 and p60R3 incorporating an *Xba*I site. The fragment *Xba*I-*Xba*I was cloned into the plasmid 2x::*GUS*, to generate the construct 4x::*GUS*. All the oligonucleotide sequences are reported in Table 1. PCR products were sequenced and the correct orientation of the fragment into the final vector was verified by restriction.

**Table 1:** Sequence of oligonucleotides used in this study

Name	Sequence
p60F1	*AAGCTT*CACAAGGACACAAGGACA
p60 F2b	*AAGCTT*CAAGTTGCAGTGAATGA
p60F8b	*AAGCTT*TAACGAGCTCCTTTTATGG
p60F9	*AAGCTT*CCATTTATGAGTTGATTATCA
p60F3	*AAGCTT*CGTGTGGAGATCAACAT
p60F5	*AAGCTT*GCAGAGTGACTCGTGA
p60R5	TCTC*GGATCC*TCTAGATCTCTCTG
p60R6	*TCTAGA*GAAGAACTTTTAAATCTGC
p60R7	*TCTAGA*AAATCTGCTTTTTCTTGAC
p60R5b	*AAGCTT*CTTTTCCATTAACTATTTTG
p60F11	*TCTAGA*CGTGTGGAGATCAACAT
p60R3	*TCTAGA*CTTTTCCATTAACTATTTTG
35SXba	*TCTAGA*CAAGACCCTTCCTC
35SBam	*GGATCC*TCCTCTCCAAATGA
mp60DOF1F1	AGTTAATGGcgcgaGCAGAGTGACTCGTGA
mp60DOF2F1	TGGCAGATCCcgcgaAGGTTGTCAAGAAAA
mp60DOF3F2	TGTCAAGAcgcgaCAGATTTAAAAGTTCTT
mp60DOF4F2	CAAGAAAAAGCAGATTTcgcgaTTCTTC
mp60WRKYF1	*AAGCTT*CGTGTGGAGATCAACATATCTTCGTTAATTGAaTACGCAAAATA
GUSRTF1	TACGGCAAAGTGTGGGTCAATAATCA
GUSRTR1	CAGGTGTTCGGCGTGGTGTAGAG
ATACT2F	TGCTTCTCCATTTGTTTGTTTC
ATACT2R	GGCATCAATTCGATCACTCA
qRT-MYB60-F	CATGAAGATGGTGATCATGAGG
qRT-MYB60-R	TTCCATTTGACCCCCAGTAG
PP2a-F	CAGCAACGAATTGTGTTTGG
PP2a-R	AAATACGCCCAACGAACAAA

Italic and lower case letters indicate restriction and mutagenised sites, respectively

### Site-directed mutagenesis analysis

Base mutations of the different DOF sites were generated using the megaprimer method [42]. For the mutagenised versions of the *AtMYB60 *minimal promoter different megaprimers were PCR amplified from plasmid p1.3-2.2::*GUS*, using as forward primers mp60DOF1F1, mp60DOF2F1, mp60DOF3F2 and mp60DOF4F2 and the single reverse primer p60R5. The megaprimers were gel purified and used in a second PCR reaction on plasmid p1.3-2.2::*GUS *with the primer p60F3. The PCR products were cloned into pCR4-TOPO and sequenced before cloning into pBI101.3 vector using the restriction sites *Hin*dIII and *Bam*HI to generate the following constructs: *mDOF1*::*GUS*, *mDOF2*::*GUS*, *mDOF3*::*GUS*, *mDOF4*::*GUS*. To generate multiple mutagenised sites the templates for the second PCR amplification were plasmids already carrying a first mutagenised DOF site. In the case of the preparation of the construct *mW*::*GUS *the megaprimer method was not necessary, as the site to mutagenise is in a position 5' terminal into the minimal promoter and a single PCR reaction was performed with primers mp60WRKYF1 and p60R5, the PCR product was then cloned with the procedure already described.

All the oligonucleotide sequences are reported in Table 1.

### Arabidopsis transformation and growth conditions

Wild-type Columbia (Col-0) plants were transformed using the *Agrobacterium tumefaciens *strain GV3101 carrying the constructs described above with the floral dip method [43]. Transformed lines were selected on kanamycin and single-insertion lines were selected for further analyses. Analyses of transgenic lines were performed on T2 or on homozygous T3 plants grown under long-day conditions (16 hr light; 8 hr darkat 100 μmol m^-2 ^sec^-1^) at 22°C in a growth chamber. Seeds were germinated in Petri dishes containing Murashige and Skoog medium, 1% w/v sucrose and 0.8% w/v agar for seedling analysis or directly on soil for adult plant organ analysis. The ABA treatment was performed as previously described [19].

### GUS activity assays and histochemical staining

For detection of GUS activity, tissues were fixed for 2 h in 90% (v/v) acetone at -20°C, incubated for 16-48 hours, at 37°C, in 0.05% (w/v) X-glucoronic acid, 0.1% (v/v) Triton X-100, and 0.5mM ferrocyanidine in 50 mM phosphate buffer (pH 7) and subsequently cleared in 70% (v/v) ethanol. Seedlings and flowers were cleared with a chloral hydrate:glycerol:water solution (8:1:2, v/v). Samples were examined using a Leica M205 FA stereomicroscope (Leica Microsystems GmbH Wetzlar, Germany) and a Zeiss Axiophot D1 microscope (Carl Zeiss MicroImaging, LLC Thornwood, New York, USA). Stereomicroscope images were recorded using the Leica LAS software version 2.8.1. Microscope images were recorded with an AxioCam MRc5 camera (Zeiss) using the AxioVision program (version 5.0).

### Quantification of mRNA expression

RNA isolation, reverse transcription, qRT-PCR reactions and data analysis were performed as previously described [30]. *GUS *expression was analysed using primers GUSRT-F1 and GUSRT-R1, *ACTIN2 *gene (primers ATACT2F, ATACT2R) was used as a reference for normalization. *AtMYB60 *expression in different *cdf *mutants was analysed using primers qRT-MYB60-F and qRT-MYB60-R. *PP2A *gene, corresponding to *At1g13320 *(primers PP2a-F and PP2a-R) was used as a reference for normalization [44]. All primer sequences are reported in Table 1.

## Authors' contributions

EC carried out the construction of promoter-reporter plasmids, plant transformation and drafted the manuscript. EC, AA and LC did transgenic Arabidopsis analysis. FF carried out *cdf *mutant analysis. CT, MG, and GC conceived the study, participated in its design and coordination. MG, LC and FF helped to draft the manuscript. All authors read and approved the final manuscript.

## Supplementary Material

Additional file 1 **Analysis of GUS activity in seeds at different developmental stages in 1,307::*GUS *line**. A: open silique showing signal only in stomata and not in developing seeds. B: mature-green-stage seed (13 DAP). C: a 24 h imbibed seed. D: embryo isolated from a 24 h imbibed seed. The same results were obtained in all transgenic lines described in Figure 2. Scale bars represent 0.1 mm. Click here for file

Additional file 2 **Relative expression of the *AtMYB60 *gene in the different *cdf *mutants**. *cdf1-R *is an RNAi line ([29]). The other single and multiple mutants have been previously described ([30]). The *PP2a *(*At1g13320*) gene was used as a control [44]. Click here for file
