# Supplementary figures and images for: DOF-binding sites additively contribute to guard cell-specificity of AtMYB60 promoter
Source: BMC Plant Biol. 2011 Nov 16;11:162. doi: 10.1186/1471-2229-11-162 (PMC3248575; doi:10.1186/1471-2229-11-162)

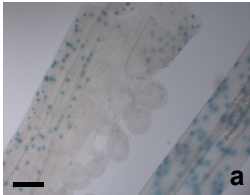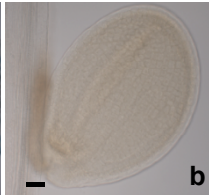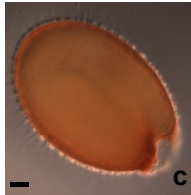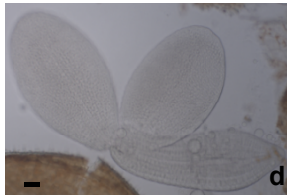

Supplement: Additional file 1 — Analysis of GUS activity in seeds at different developmental stages in 1,307::GUS line. A: open silique showing signal only in stomata and not in developing seeds. B: mature-green-stage seed (13 DAP). C: a 24 h imbibed seed. D: embryo isolated from a 24 h imbibed seed. The same results were obtained in all transgenic lines described in Figure 2. Scale bars represent 0.1 mm. [file 1471-2229-11-162-S1.PDF]

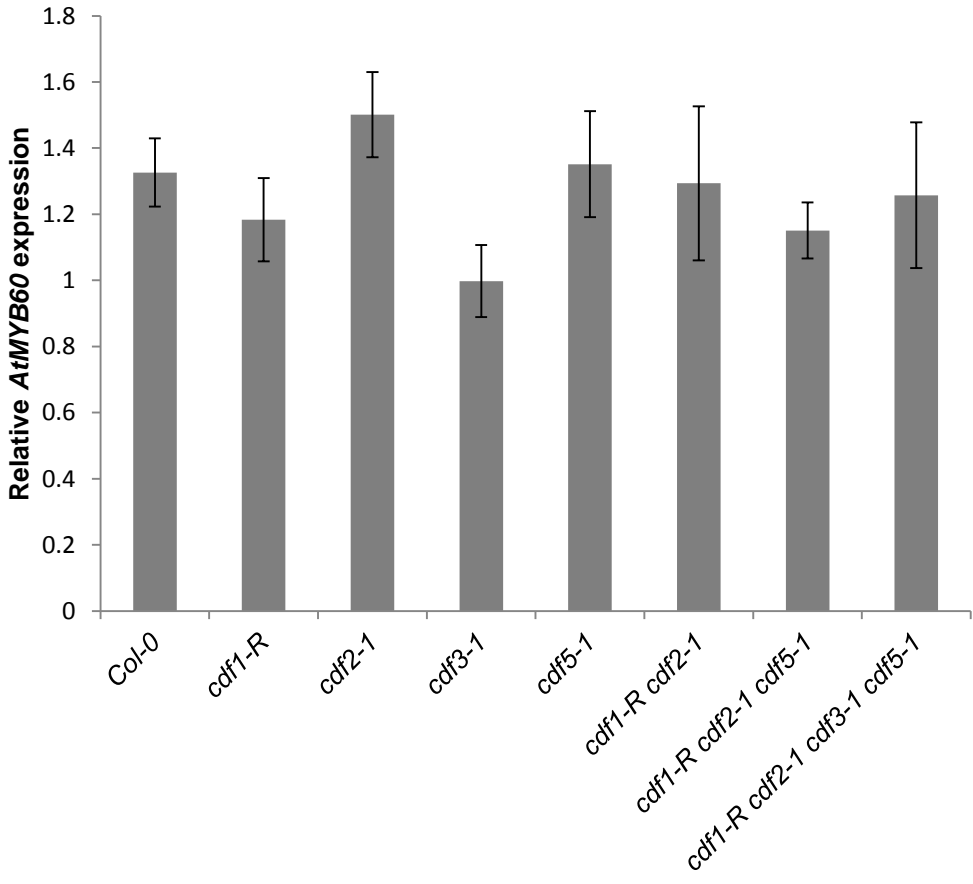

Supplement: Additional file 2 — Relative expression of the AtMYB60 gene in the different cdf mutants. cdf1-R is an RNAi line ([29]). The other single and multiple mutants have been previously described ([30]). The PP2a (At1g13320) gene was used as a control [44]. [file 1471-2229-11-162-S2.PDF]
